# Supplementary figures and images for: Mortality and evolution between community and hospital-acquired COVID-AKI
Source: PLoS One. 2021 Nov 4;16(11):e0257619. doi: 10.1371/journal.pone.0257619 (PMC8568145; doi:10.1371/journal.pone.0257619)

**Figure 1 Appendix.** Overall, 28-days survival in hospitalized COVID patients.


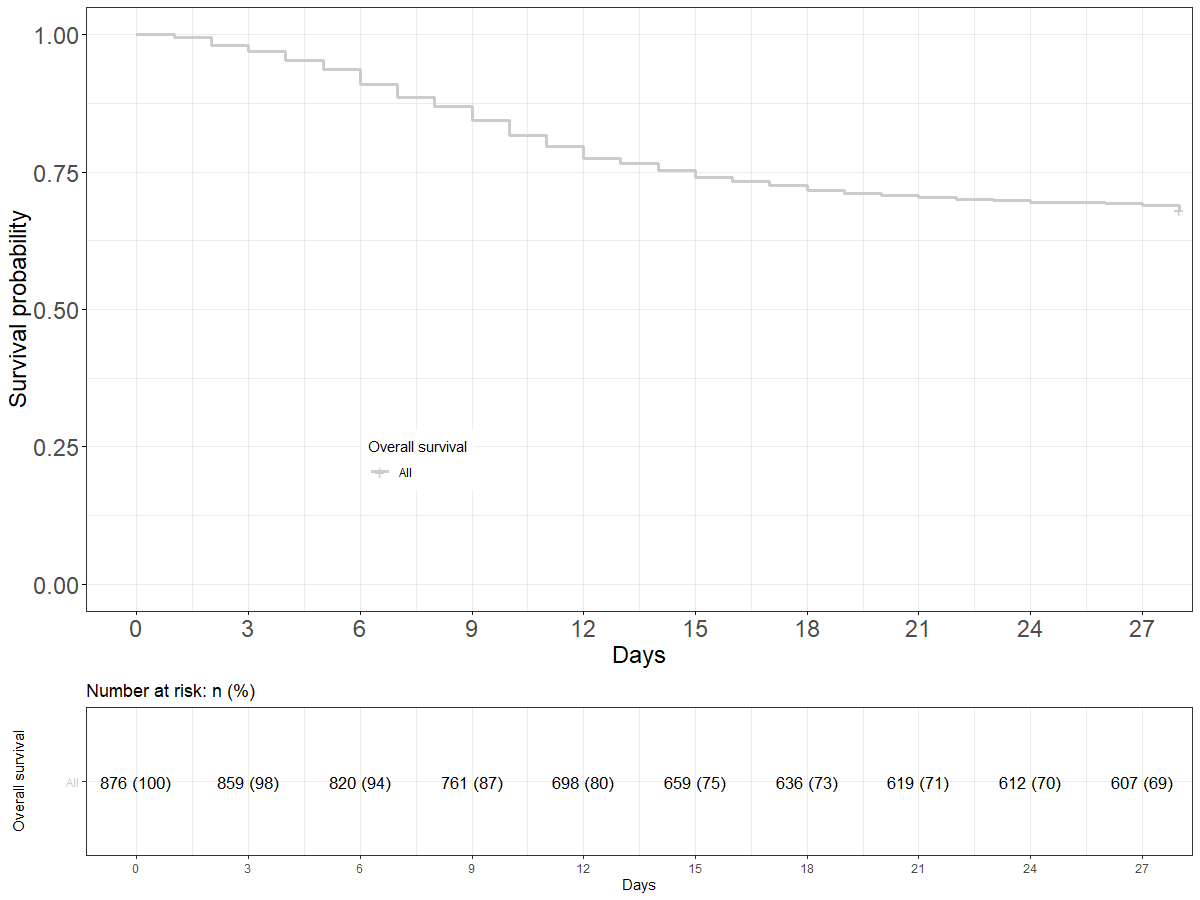

Supplement: S1 Fig — (DOCX) [file pone.0257619.s001.docx]
