# Supplementary material for: Mortality and evolution between community and hospital-acquired COVID-AKI
Source: PLoS One. 2021 Nov 4;16(11):e0257619. doi: 10.1371/journal.pone.0257619 (PMC8568145; doi:10.1371/journal.pone.0257619)

**Figure 2 Appendix.** Kaplan–Meier survival statistics comparing AKI stages and mechanical ventilation. Numbers of patients at risk at each time point shown below the graph.


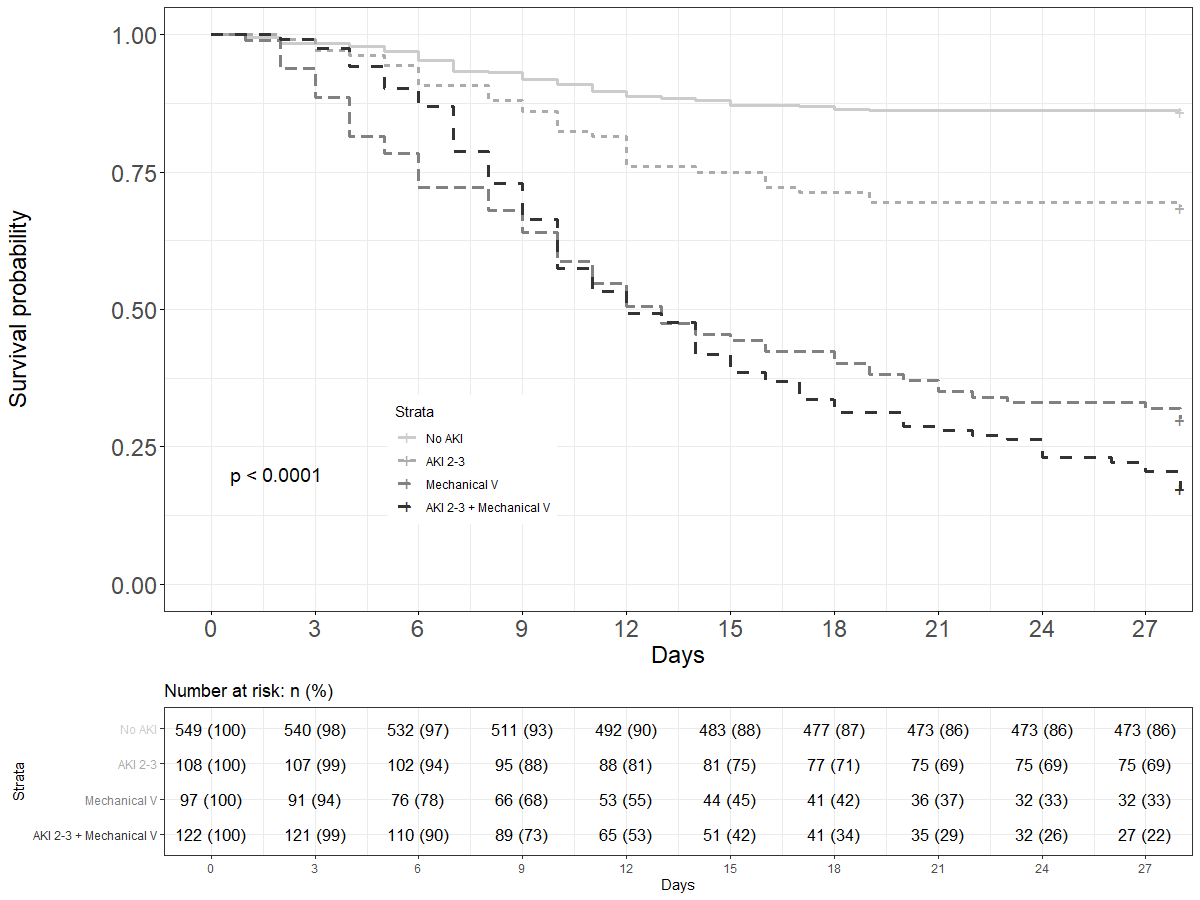

Supplement: S2 Fig — Numbers of patients at risk at each time point shown below the graph. (DOCX) [file pone.0257619.s002.docx]
