# Supplementary material for: Mortality and evolution between community and hospital-acquired COVID-AKI
Source: PLoS One. 2021 Nov 4;16(11):e0257619. doi: 10.1371/journal.pone.0257619 (PMC8568145; doi:10.1371/journal.pone.0257619)
Supplement: Attachment — Submitted filename: response to reviewer 25_6_21.docx [file pone.0257619.s004.docx]

**Response to Reviewers'**

We appreciate the time and the review process, without a doubt the comments of the editors are all relevant and pertinent, they contribute to making our manuscript stronger. We have done our best to satisfy each of them.

**Reviewer #1:**

(1) Please provide additional details regarding participant consent. In the ethics statement in the Methods and online submission information, please ensure that you have specified what type you obtained (for instance, written or verbal, and if verbal, how it was documented and witnessed). If your study included minors, state whether you obtained consent from parents or guardians. If the need for consent was waived by the ethics committee, please include this information.

r= we add the phrase:

Written informed consent was obtained from all of the subjects at hospitalization in the methods section

(2) Please upload a copy of Figure 5 which you refer to in your text on page 23. Or if the figure is no longer to be included as part of the submission please remove all reference to it within the text.

R= There was a typographical error, there is no figure 5, we change the number 4 to 5 in the text.

(3) Please include a legend for Figure 5.

R= There was a typographical error, there is no figure 5, we change the number 4 to 5 in the text.
