# Supplementary material for: Mortality and evolution between community and hospital-acquired COVID-AKI
Source: PLoS One. 2021 Nov 4;16(11):e0257619. doi: 10.1371/journal.pone.0257619 (PMC8568145; doi:10.1371/journal.pone.0257619)
Supplement: Attachment — Submitted filename: response to Reviewer 30_7_21.docx [file pone.0257619.s005.docx]

**Authors response to the reviewers**

We appreciate the excellent work of the reviewers, without a doubt their comments and opinions have been of great value to us, they add greater quality to the manuscript. We humbly hope that our responses will satisfy your requests.

**Reviewer #1**: **The authors have revised the document, but there remains inherent inconsistencies with the data.**

**Authors have 230 patients who did not have baseline within 3 months and were excluded. Did these patients have baseline creatinine in last 12 months? For other patients, baseline creatinine within a year was considered and there is no clear explanation offered by the authors.**

R= These 230 patients who were excluded was because lack of data prior to hospitalization, or who for some reason were not taken in hospitalization. We add in figure 1, point 5, the phrase lack of data.

**The manuscript still has conflicting use of mortality and survival Eg: page 14**

R=We changed the word mortality to survival in page 14.

Survival was used when we reported overall survival, the 28-day survival comparing CA-AKI, HA-AKI, and mechanical ventilation (Fig 2), and the 28-day survival between AKI stages and mechanical ventilation (S2 Fig).

Mortality was used when we reported the percentage of death among those with different types of AKI (Fig 3), and in the multivariable analysis of variables associated with death (Table 3).

**The study is retrospective observational study and consent was taken at the time of hospitalization. The conclusion states that this is prospective study.**

R=The data were recruited prospectively, from the hospitalization of the patients at the time of admission, informed consent was obtained for the storage of information. In the abstract and methods section, we change the word retrospective to prospective.

**There are too many unnecessary tables. On page 14, please ensure confidence interval is correctly reported.**

R= Done. We check the confidence intervals, and they are correct.

**Kaplan Meier curve has time on x-axis, which does not make sense and should have days. Even with days, Kaplan Meier curve is inappropriate for short duration of follow-up of 28 days and does not add any information on long–term outcome.**

R= The Kaplan-Meier procedure is a method of estimating time-to-event models in the presence of censored cases. The Kaplan-Meier model is based on estimating conditional probabilities at each time point when an event occurs and taking the product limit of those probabilities to estimate the survival rate at each point in time.
We understand that the follow-up time is not very long compare to other long-term diseases but due to the severity of the disease that is being analyzed here, the use of a kaplan-meier analysis with a 28-day follow-up is adequate, not to mention that it also offers visual support for greater understanding of the reader.

We also add a new supplemental material file with the figures change in DAYS instead of TIME.

**Reviewer #2: Dear authors,
The manuscript makes a different contribution to the understanding and management of patients with AKI-Covid.**

**I highlight the data on the mortality score for AKI-Covid. However, the manuscript needs adjustments:**
**Abstract
Use the KDIGO standardized nomenclature for renal replacement therapy-RRT (throughout the text) instead of KRT.**

R= In the last KDIGO conference report 2020 it was attended to use “kidney” instead of “renal” or “nephro-“. They suggested to use “Kidney replacement therapy” and to avoid “Renal replacement therapy”.

Kidney International (2020) 97, 1117–1129; https://doi.org/10.1016/ j.kint.2020.02.010

**Conclusions: add data on mechanical ventilation and AKI.**
Introduction

R= We add 6 more references about the strong association between mechanical ventilation and AKI, you can find them in page 20

**Subsidize with more references. Sugestions:**
**https://pubmed.ncbi.nlm.nih.gov/32416769**
[**https://pubmed.ncbi.nlm.nih.gov/34033655**](https://pubmed.ncbi.nlm.nih.gov/34033655)

R= We appreciate the suggestions of these 2 references, we include interesting information about them in the Discussion, in addition, we add 2 additional ones. You can find them with the number in pages 18, 19 and 20

**Methods
I would like to see the ethics committee protocol number.**

R= we add the committee protocol number in methods page 5.

The Institutional Review Board of both hospitals (Hospital Civil de Guadalajara HCG/CEI-0473/20 and Centro Medico Nacional 20 de Noviembre 09-240.2020)

**Results
Format the tables. They are presented in frame format.**

R= done, tables are presented in frame format.

**I suggest unifying the results of tables 3 and 4; 5 and 6.**

R= Done. Thanks for the suggestion, tables 3 and 4 ; 5 and 6 were unifying, in there would be only table 3 and 4. You can find them in pages 15, 16 and 17.

**Present the score data only with figure 4. Leave table 7 in the supplementary material.**

R= Done.

**Figure 1 is incomplete. Please enter the subdivisions of patients who were elected in the study.**

R= done, we included elected patients (in the first square)

**Discussion**

**Increase the number of references.**

R= We appreciate the suggestions. We found articles that we consider interesting to include in our manuscript, they were essentially added to the Discussion. You can find in page 17-21.

**Better discuss the findings
For example: The Body Mass Index was high and although without statistical relevance in your study, it has been observed that overweight and obesity are risk factors for covid-19.**

R=We understand the point of discussing the BMI as an interesting point, we appreciate that you have pointed it out. We add reference 25 (a meta-analysis on the relationship between obesity and COVID) and contrast our findings with this variable in the discussion, we believe that it certainly adds value to our manuscript.
