# Supplementary material for: Mortality and evolution between community and hospital-acquired COVID-AKI
Source: PLoS One. 2021 Nov 4;16(11):e0257619. doi: 10.1371/journal.pone.0257619 (PMC8568145; doi:10.1371/journal.pone.0257619)
Supplement: Attachment — Submitted filename: Response to Reviewers 16_8_21.docx [file pone.0257619.s006.docx]

**Response to reviewers**

We deeply appreciate the comments of the reviewers and are pleased that their requests have been successfully fulfilled.

We have made the 2 changes suggested by the editorial committee.

- **Please correct the titles of the tables "Univariable-Multivariable logistic regression..." to better readers' perception, and use the coherent terminology in the title and the column names (Univariate/Multivariate)**

R= Done, titles of Table 2 and 3 of the words "Univariable-Multivariable" by "Univariate / Multivariate"

**-Please exclude subtitles "-Mortality of CA-AKI vs HA-AKI: ", etc from the "Discussion", changing them with a short introductory phrase.**

R= Done, we exclude subtitles "-Mortality of CA-AKI vs HA-AKI:", "Risk factors for CA-AKI", "KRT between CA-AKI vs HA-AKI" and "Predictive score of death in patients with COVID-AKI" from the "Discussion". We believe that only by deleting those subtitles and leaving the first sentence of each paragraph as it is in the manuscript, the content is perfectly understood, without adding anything else.
