# Supplementary material for: Mortality and evolution between community and hospital-acquired COVID-AKI
Source: PLoS One. 2021 Nov 4;16(11):e0257619. doi: 10.1371/journal.pone.0257619 (PMC8568145; doi:10.1371/journal.pone.0257619)
Supplement: Attachment — Submitted filename: Response to Reviewers 21_8_21.docx [file pone.0257619.s007.docx]

**Response to reviewers**

We deeply appreciate the comments of the reviewers and are pleased that their requests have been successfully fulfilled.

We have welcomed comments from the Academic Editor Boris Bikbov about minor edits to the tables and text, which we have corrected according to their instructions. Now the manuscript is easier to read.

**"Table 3. Univariable/Multivariable logistic regression model to determine the variables associated with mortality at 28-days follow up and Kidney replacement therapy in hospitalized COVID patients." could be changed to "Table 3. Factors associated with mortality at 28-days of follow up and the requirement of kidney replacement therapy in hospitalized COVID patients, in the univariable and multivariable logistic regression model." This is just an example, you could change the titles in your own way, but I hope this example demonstrates the idea.**

R=Done, The changes have been made in table 3 and 4 with the editor's suggestions, now it is as follows:

Table 3. Factors associated with mortality at 28-days of follow up and the requirement of kidney replacement therapy in hospitalized COVID patients, in the univariable and multivariable logistic regression model. (page 16)

Table 4. Factors associated with CA-AKI and HA-AKI in hospitalized COVID patients, in the univariable and multivariable logistic regression model. (page 17)

**Please also change the terminology used in the table ("Grade COVID" -> "COVID severity", "Mechanical ventilator" -> "Need in mechanical lung ventilation") and also in the manuscript text ("ventilator" -> "ventilation", etc).**

R= Done, we have made the changes suggested by the Editor in tables and the manuscript, of the terms: grade COVID, mechanical ventilator; by the terms: COVID severity, Need in mechanical lung ventilation.

We really hope that this changes satisfy to the Academic Editor.
